# Differences in Lipid Measurements by Antiretroviral Regimen Exposure in Cohorts from Asia and Australia

**DOI:** 10.1155/2012/246280

**Published:** 2012-05-14

**Authors:** Amit C. Achhra, Janaki Amin, Jennifer Hoy, Junko Tanuma, Thira Sirisanthana, David Nolan, Tuti Merati, Michelle Giles

**Affiliations:** ^1^The Kirby Institute for Infection and Immunity in Society (Formerly the National Centre in HIV Epidemiology and Clinical Research), Faculty of Medicine, University of New South Wales, Sydney, NSW 2052, Australia; ^2^Infectious Diseases Unit, Alfred Hospital and Monash University, Melbourne, VIC 3004, Australia; ^3^AIDS Clinical Center, National Center for Global Health and Medicine, Tokyo 162-8655, Japan; ^4^Research Institute for Health Sciences, Chiang Mai University, Chiang Mai 50200, Thailand; ^5^Centre for Clinical Immunology and Biomedical Statistics, Murdoch University and Royal Perth Hospital, Perth, Australia; ^6^Faculty of Medicine, Udayana University and Sanglah Hospital, Bali 80233, Indonesia

## Abstract

We explored the mean differences in routinely measured lipids (total cholesterol, triglycerides, and high-density lipoprotein cholesterol) according to exposure to different combination antiretroviral regimens in Asian (*n* = 2051) and Australian (predominantly Caucasian, *n* = 794) cohorts. The regimen was defined as at least 3 antiretroviral drugs with at least 2 nucleoside-reverse transcriptases (NRTIs) and either of at least one protease inhibitor (PI) or non-nucleoside-reverse transcriptases (NNRTIs). We categorised cART regimens as: NRTIs as tenofovir based or not; NNRTIs as nevirapine or efavirenz (but not both); and PI as atazanavir based or not. We found that the impact of various antiretroviral regimens on lipids in Asian and Australian cohorts was only different by cohort for total cholesterol (*P* for interaction between regimen and cohort: <0.001) but not in case of other lipids (*P* for interaction: >0.05). The differences in total cholesterol were however small and unlikely to be of clinical significance. Overall, tenofovir with nevirapine or atazanavir was associated with the most favorable lipids, while the PI regimens without tenofovir and atazanavir were associated with least favorable lipids. We conclude that the impact of various ART regimens on lipids is largely similar in Asian and Australian cohorts and that the newer drugs such as tenofovir and atazanavir are likely to provide similar benefit in terms of lipid profiles in both populations.

## 1. Introduction

Combination antiretroviral therapy (cART) for HIV infection is associated with adverse changes in lipid profiles and can include elevation in total cholesterol and triglycerides, which may increase the risk of coronary heart disease (CHD) [1–4]. Moreover, different classes of cART and drugs within each class have differential impacts on lipids [2]. Protease-inhibitors (PIs) are associated with more significant changes in lipid profile than nucleoside and nonnucleoside reverse transcriptase inhibitors (NRTIs and NNRTIs, resp.) [2, 3, 5]. And within NNRTI class, efavirenz (EFV) is associated with greater changes in the lipid profile than nevirapine (NVP) [2, 5, 6]. Also tenofovir (TDF) and atazanavir (ATV) are known to have a favorable impact on lipids [5, 7, 8].

Drugs such as TDF, EFV, and ATV are becoming increasingly available in low-middle-income countries, including Asia [9, 10]. However, much of our knowledge about the relative impact of different cART regimens on lipids comes mainly from clinical trials and cohort studies from European or North-American settings [2, 4, 7, 8]. The impact of cART on lipids may vary in Asian settings due to differences in race/ethnicity, dietary, environmental, and lifestyle factors [11–13]. This has been demonstrated in other settings where the magnitude of change in total cholesterol and triglycerides due to PIs differed between African Americans and Caucasians, highlighting the possible role of race [11, 12]. These findings illustrate the need for verifying our assumptions about the relative impact of different cART regimens on diverse populations, including Asian populations.

Observational cohort studies can complement information from clinical trials, and allow us to examine the effects of art medications in the context of combination regimens, as opposed to head-to-head comparisons of selected drugs in clinical trials. In the present study, we aim to compare the relative impact of various cart regimens on lipid profiles in Asian and Australian cohorts using data from the treat Asia and the Australian HIV observational databases (TAHOD and AHOD, resp.), which are formed on similar methodology and are known to be predominantly Asian and Caucasian, respectively [14].

## 2. Methods

### 2.1. The TAHOD and AHOD Cohorts

TAHOD and AHOD are clinical cohort studies of HIV-infected patients in Asia and Australia, respectively, and are part of the International Epidemiologic Databases to evaluate AIDS initiative. Both cohorts have similar methodologies, which have been previously published [15, 16]. Briefly, prospective data collection was commenced in 2003 for TAHOD and in 1999 for AHOD, with retrospective data being provided where available. In TAHOD, data are collected from 17 clinical sites in the Asian region, whereas for AHOD, data are collected from 27 clinical sites throughout Australia. Written informed consent was not a requirement of sites in TAHOD unless required by the site's local ethics committee because data are collected in an anonymous form, while in AHOD consent was obtained from all patients recruited at the time of enrolment. The TAHOD and AHOD cohorts are known to be predominantly of Asian and Caucasian ethnic composition, respectively [14].

Ethical approval for both the cohorts was obtained from the University of New South Wales, Sydney, Australia, and all other relevant institutional review boards. Data for both TAHOD and AHOD are transferred electronically to the Kirby Institute twice per year and include the same set of core variables. All data are subject to standardized quality control procedures.

### 2.2. Outcome

The outcomes of interest were mean (i) total cholesterol, (ii) triglycerides, (iii) high-density lipoprotein cholesterol (HDL-C) measured in mmol/L, and (iv) total cholesterol : HDL-C ratio. Lipid values are measured according to the local sites' standard of care in each cohort, and when measured, are captured during routine data transfer. TAHOD only records fasting lipids. In AHOD, both fasting and nonfasting lipids along with the fasting status are recorded. Further details of laboratory standards and methods at each site were not available.

Data collection on lipid profiles started later in AHOD (median date: January, 2007), compared to TAHOD (median date: March, 2006). The lipid values before starting cART (i.e., while patients were ART naive) were not available in most patients, and therefore changes from pre- to post-cART were not analysed. Mean lipid measurements were compared by different regimens and cohort.

### 2.3. Definition and Classification of Antiretroviral Regimens

The cART regimen variable was defined as a regimen containing at least 3 antiretroviral drugs, including at least two NRTIs and either of at least one PI or an NNRTI. In order to evaluate the net effect of a combination regimen, rather than that of a single drug or a class, we defined eight mutually exclusive regimens. We categorised cART regimens as: NRTIs as TDF based (NRTIs + TDF) or not (NRTIs); NNRTIs as NVP or EFV (but not both); and PI as ATV based (PI + ATV) or not (PI).

Based on these categories, the following mutually exclusive regimens were defined: (i) NRTIs (+TDF) + NVP; (ii) NRTIs (+TDF) + EFV; (iii) NRTIs + NVP; (iv) NRTIs + EFV; (v) NRTIs (+TDF) + PIs (+ATV); (vi) NRTIs (+TDF) + PI; (vii) NRTIs + PI (+ATV); (viii) NRTIs + PI. In all analyses, regimen (i) NRTIs (+TDF) + NVP was used as the reference group, as this regimen was thought to have the most favourable impact on lipids.

### 2.4. Inclusion Criteria and Time-Points Analysed

Patients from TAHOD and AHOD were eligible for inclusion in the analysis if they started cART and had at least one lipid measurement within the first 24 months of cART commencement. Time at risk was defined as time spent on any of the regimens described previously and risk time started from the commencement of that regimen. Follow-up was censored at first of 24-month exposure to regimen of interest, date of death, loss to follow-up, or 31 March, 2010. Lipids values measured at the 6-monthly intervals in the first 24 months of start of cART were used. Thus each patient on each regimen could have up to 4 measurements (1 in each interval). If more than one measurement was available in a given interval, one measured earliest in the given interval was used in the analysis. Intermittent changes in therapy including stopping part or all of a regimen for less than 14 days were not considered a stop in time at risk for that regimen. Each patient could contribute data to more than one regimen.

### 2.5. Variables and Statistical Analysis

The following *a priori* confounders were included in all models:

1. fixed variables: cohort (TAHOD/AHOD), gender, HIV transmission group (homosexual contact ± intravenous drug user (IDU), IDU ± heterosexual, heterosexual, and other), and hepatitis B and C coinfection (defined as HBV surface antigen and HCV antibody positive, resp.);
2. variables measured closest to the start of each cART regimen within past 6 months to 1 month after the start of the regimen of interest: CD4+ T-cell count (categorised as <200, 200–350, and >350 cells/*μ*L); HIV RNA viral load (categorised as <500, >500–<10,000, and >10,000 copies/mL); and body mass index (BMI) (categorised as <18.5, 18.5–25, 25–30, and >30 kg/m^2^);
3. variables recalculated at the start of each cART regimen: cumulative cART exposure and age.

We performed longitudinal data analysis using random effects models to take into account repeated lipid measures (defined previously). Since all lipid parameters were normally distributed with minimal skewness, data were not transformed. Separate models were fitted for each outcome. All models included time on regimen with lipid data, categorised as 6 monthly intervals. The interaction between the regimen and the cohort variables was assessed for each outcome. We also conducted the following sensitivity analyses: (i) restricting AHOD data to only lipid values which were documented to be taken as fasting in AHOD, (ii) excluding patients with missing BMI data, (iii) including only those with known Caucasian ethnicity in AHOD, and (iv) additional adjustment for stavudine (d4t) use in the multivariable model, as it was more common in TAHOD than AHOD.

Data were analysed using STATA version 10 (STATA Corporation, College Station, TX, USA).

## 3. Results

There were 2845 participants (2051 in TAHOD and 794 in AHOD) who met the inclusion criteria. In TAHOD, 736 (35.9%) were Chinese, 654 (31.9%) were Thai, 152 (7.4%) were Cambodian (Khmer), 100 (4.9%) were Japanese and 62 (3%) were Indian.  Table 1 describes the patient characteristics at study entry for each cohort.

There were a total of 7897 total cholesterol values (5602 in TAHOD and 2295 in AHOD), 7293 triglyceride values (5002 in TAHOD and 2291 in AHOD), and 4669 HDL-C values (2949 in TAHOD and 1720 in AHOD). The frequency of total cholesterol measurements by regimen and cohort is shown in Table 2. The most common NRTI combinations for which total cholesterol measurements were available in TAHOD were zidovudine (AZT)/lamivudine (3TC) (39% of all measurements) and d4t/3TC (30% of all measurements) and in AHOD TDF/emtricitabine (FTC) (29% of all measurements) and abacavir (ABC)/3TC (18% of all measurements). The distribution was similar for triglycerides and HDL-C. In TAHOD and AHOD, 52% and 46% of measurements were taken while on NNRTI-based regimens, respectively. Of all the measurements on PI-based regimens, greater than 95% were on ritonavir-boosted regimens.

Patients contributed data to the median of 1 regimen (range: 1 to 4) with a median of 2 lipid measurements (IQR: 1–4) per patient. All measures of CD4 cell count, HIV viral load, and BMI were collected within 35 days of commencing the different ART regimens. Participants from TAHOD were more likely to be younger and female and have heterosexually acquired infection, hepatitis B coinfection, detectable HIV VL, lower median CD4+ count, shorter time spent on cART, lower BMI, lower mean total cholesterol, and higher HDL-C than those from AHOD (Table 1). Also a higher proportion of TAHOD participants had missing BMI and HIV viral load values compared with those in AHOD (Table 1).

### 3.1. Total Cholesterol

**Figure 1:**
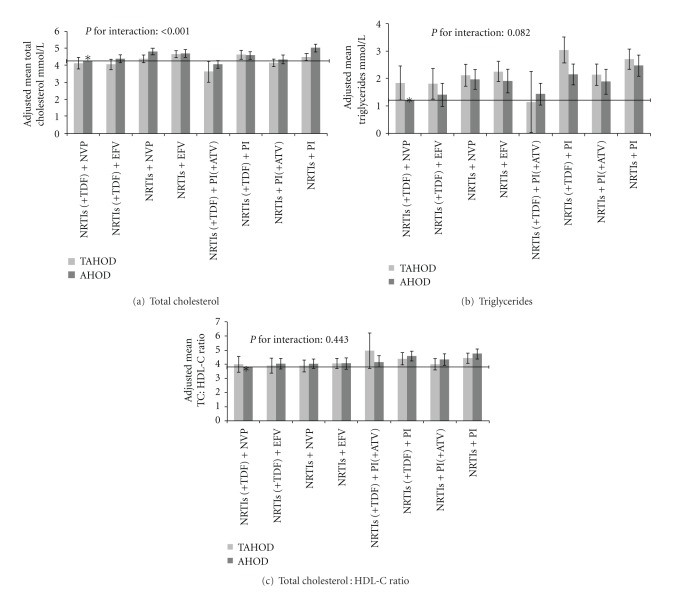
Adjusted Mean Lipids by regimen and cohort. Interaction between impact of regimen and cohort variables on lipids: (a) mean total cholesterol, (b) mean triglycerides, and (c) mean total cholesterol : HDL-C ratio. A statistically significant interaction suggests impact of regimen on lipids differed in magnitude by the cohort. Triglycerides were higher for TAHOD as compared to AHOD irrespective of the regimen, but the interaction between regimen and cohort was not significant. Means were *a priori* adjusted for time on given regimen, HBV and HCV confections, age, gender, HIV RNA viral load copies/mL, CD4+ T-cell count, BMI, cumulative exposure to cART at baseline, and HIV exposure category. *Reference category. Horizontal line shows the value of constant (mean lipid value in reference category). Error bars indicate 95% confidence interval. AHOD: Australian HIV Observational Database; ATV: Atazanavir; EFV: Efavirenz; NNRTIs: nonnucleoside reverse transcriptase inhibitors; NRTI: Nucleoside reverse transcriptase inhibitors; NVP: Nevirapine; PI: protease inhibitor; TAHOD: TREAT Asia HIV Observational Database; TDF: Tenofovir. *Key*. NRTIs are TDF based (NRTIs + TDF) or not (NRTIs) and PI as ATV based (PI + ATV) or not (PI).

The relationship between mean total cholesterol and cART regimen differed by cohort (TAHOD/AHOD) (*P* < 0.001, test for interaction). Overall, the mean total cholesterol was slightly lower for TAHOD participants, compared to AHOD participants, after adjustment for demographic and HIV-related characteristics, in most of the regimens (Figure 1(a)). When compared to the NRTIs (+TDF) + NVP regimen (reference group), the NRTIs + PI regimen was associated with greater mean total cholesterol in both cohorts, with a slightly greater difference in AHOD participants (mean difference: +0.78 mmol/L, 95% CI: 0.57 to 1.00) compared to TAHOD participants (mean difference: +0.23 mmol/L, 95% CI: 0.02 to 0.44); NRTIs (+TDF) + PI (+ATV) regimen was associated with greater mean total cholesterol in AHOD (mean difference: −0.20 mmol/L, 95% CI: −0.43 to 0.02) as compared to TAHOD (mean difference: −0.62 mmol/L, 95% CI: −1.23 to −0.02).

### 3.2. Triglycerides, HDL-C, and Total Cholesterol: HDL-C Ratio

There was no significant interaction between cART regimen and the cohort type (*P* > 0.05, test for interaction) for triglycerides, HDL-C, and total cholesterol: HDL-C ratio.  Table 3 provides adjusted analyses for each of these outcomes. As compared to the NRTIs (+TDF) + NVP regimen (reference group), the NRTIs + PI regimen was associated with the highest mean triglycerides (mean difference: 1.13 mmol/L, 95% CI: 0.83 to 1.43) and total cholesterol : HDL-C ratio (mean difference: 0.75, 95% CI: 0.47 to 1.03), followed by the NRTIs (+TDF) + PI regimen (mean difference in triglycerides: 1.06 mmol/L, 95% CI: 0.73 to 1.38, and mean difference in total cholesterol : HDl-C ratio: 0.66, 95% CI: 0.37 to 0.95), while NRTIs (+TDF) + PI (+ATV) regimen was not associated with a significant difference in triglycerides (mean difference: 0.15 mmol/L, 95% CI: −0.22 to 0.52) and total cholesterol : HDL-C ratio (mean difference: 0.29, 95% CI: −0.04 to 0.62). Also, the NRTIs + EFV regimen was associated with increase in triglycerides (mean difference: 0.64 mmol/L, 95% CI: 0.34 to 0.95) and total cholesterol : HDL-C ratio (mean difference: 0.29, 95% CI 0.01 to 0.56). The TAHOD cohort, as compared to AHOD, had higher mean triglycerides, but not total cholesterol : HDL-C ratio. Figures 1(b) and 1(c) provide the graphical representation of the adjusted mean triglycerides and total cholesterol : HDL-C ratio for each regimen and cohort, respectively.

When compared to the reference group, the NRTIs + NVP regimen and the NRTIs + EFV regimen were associated with higher mean HDL-C (mean difference of: 0.15 mmol/L, 95% CI: 0.08 to 0.21 and 0.09 mmol/L, 95% CI: 0.03 to 0.16, resp.). Other regimens were not significantly different to the reference regimen.

### 3.3. Sensitivity Analyses

Forty-five percent of all of the AHOD measurements were taken fasting. Ethnicity was known in AHOD in 80% of participants, of whom greater than 80% were Caucasian. All of the sensitivity analyses, except for exclusion of missing BMI data, yielded very similar results, in terms of direction of effect, magnitude, and significance, as those from full analyses (data not presented). Since BMI was missing in a significant proportion of participants in both cohorts (Table 1), restriction of analysis to only patients with known BMI provided results that were of similar direction and magnitude of the effect, however in some cases less statistically significant, because of loss of power.

## 4. Discussion

In this study, we examined the mean differences in lipids between various cART regimens in TAHOD and AHOD cohorts. We found that the relationship between regimen and lipids differed by cohort only in the analysis of total cholesterol level. For total cholesterol, the magnitude of effect of regimen differed between cohorts such that TAHOD participants tended to have slightly lower total cholesterol for most regimens (most notably for the NRTIs (+TDF) + PI (+ATV) and the NRTIs + PI regimens). Overall, we found that the NRTIs (+TDF) + NVP and the NRTIs (+TDF) + PI (+ATV) regimens were associated with the most favorable lipid profile, whereas the NRTIs + PI and the NRTIs + EFV regimens were associated with the least favorable lipid profiles.

These regimen/lipid association findings are consistent with the literature from Western countries [5–8, 17]. The few studies that have reported lipid results from Asian population also suggest an adverse impact of PI-based and EFV-based regimens on lipids as compared to non-PI-based and NVP-based regimens, respectively [18, 19]. However, these studies were performed in clinical-trial populations, and in one study [19], NVP was given at 400 mg once a day, instead of the recommended 200 mg twice a day dosing schedule. Also, they did not report usage of TDF- or ATV-based regimens.

The observed differences in mean total cholesterol between TAHOD and AHOD cohorts, though statistically significant, were of small magnitude. The differences could be due to variations in race/ethnicity or dietary, environmental, and lifestyle factors [20]. The clinical relevance of these differences, particularly the impact of these differences on overall risk of CHD, is uncertain. In our study, TAHOD participants had average total cholesterol up to 0.5 mmol/L lower than AHOD for the NRTIs + PI regimen (Figure 1(a)). Studies on treated HIV-infected populations have suggested that a difference of 1 mmol/L in total cholesterol may be associated with difference of about 25% in risk of CHD [21, 22]. Further, TAHOD participants, for any given regimen, had higher triglycerides which have been associated with greater risk of CHD [23]. The CHD events in cART-treated HIV patients are thought to be multifactorial in origin, with a possible role of dyslipidemia, cART, HIV-associated inflammatory process, and traditional risk factors [1, 21]. We did not have data on other CHD risk factors, with which we could calculate the overall Framingham risk score for each cohort. Future studies should therefore evaluate their impact on risk of CHD events in treated HIV populations in Asia.

There are further limitations to this study. We divided each class of ART according to those shown to have favorable impact on lipids, that is, TDF, ATV, and NVP [5, 7, 8]. However, such a classification did not allow us to examine the impact of other individual drugs in each class. Further, we did not have information on use of lipid-lowering medications, which are likely to differ between TAHOD and AHOD. It is likely that since TAHOD participants were younger and mostly from low-middle income countries, they may have a lower rate of use of lipid-lowering medications, as compared to those in AHOD. However, TAHOD participants had lower total cholesterol for most regimens than those in AHOD, suggesting residual confounding, racial differences, or possibly higher use of lipid lowering medications. Also, lipid values were measured in site-specific laboratories which may introduce variation in results. However, such a variation is unlikely to result in any systematic differences in total cholesterol that we observed. Furthermore, patients in AHOD tended to be more treatment-experienced than TAHOD; however, adjusting for cumulative cART exposure did not change our results. Nevertheless, any residual confounding from these and other unmeasured factors cannot be ruled out. Lastly, we did not have pre-cART lipid data to compare with post-cART data in each cohort, which would have provided clearer comparisons of change in lipids in response to cART between the cohorts. Keeping these limitations in mind, our results were however robust when restricted to those (i) documented to be taken after over-night fasting, (ii) with documented race/ethnicity, and (iii) adjusting for differential use of d4t, suggesting that these factors were unlikely to impact our results.

Strengths of our study include TAHOD and AHOD being founded on similar methodology thereby reducing the likelihood of confounding due to methodological differences. Also, the large sample size available allowed us to analyse several regimens, including those with TDF and ATV, which are less frequently used in low-middle income countries [10]. Moreover, availability of lipid measurements on several time-points on each regimen allowed us to adjust for time spent on each regimen of interest.

In summary, our findings suggest that the impact of various ART regimens on lipids is largely similar in TAHOD and AHOD cohorts and that the newer drugs such as TDF and ATV are likely to provide similar benefit in terms of lipid profiles in both populations. We also found that TAHOD participants may have slightly lower mean total cholesterol for most regimens, although the clinical significance of this difference is uncertain. These findings contribute to the gap in evidence from Asian settings. Future studies should report pre- to post-ART monitoring of lipids and information on Framingham risk score in diverse populations.

## Figures and Tables

**Table 1:** Patient characteristics at study entry.

Characteristics	AHOD, *n* = 794 *n* (%)	TAHOD, *n* = 2053 *n* (%)	P*
Age (years)			
Mean ± SD	45 (±9.8)	38.6 (±10)	<0.001

Gender			
Male	761 (96)	1,484 (72.2)	<0.001
Female	30 (3.8)	567 (27.6)	
Transgender***	03 (0.4)	02 (0.1)	

HIV exposure category			
Homosexual contact ± IDU	618 (78.4)	485 (23.7)	<0.001
IDU ± heterosexual	25 (3.2)	45 (2.2)	
Heterosexual	65 (8.2)	1,341 (65.6)	
Other	80 (10.2)	174 (8.5)	
Missing	06 (0.7)	08 (0.4)	

HBV coinfection			
Negative	649 (81.8)	1,376 (67)	<0.001
Positive	22 (2.8)	171 (8.3)	
Missing/never tested	123 (15.5)	506 (24.6)	

HCV coinfection			
Negative	631 (79.5)	1,365 (66.5)	<0.001
Positive	87 (11)	128 (6.2)	
Missing/never tested	76 (10)	560 (27.3)	

HIV RNA < 500 copies/mL	326 (41)	372 (18)	<0.001
Missing	58 (7.3)	880 (43)	

CD4+ count cells/*μ*L	360 (219–569)	161 (50–280)	<0.001**
Median (IQR)			
Missing	58 (7.3)	264 (12.9)	

Cumulative exposure to cART in years			
Median (IQR)	2 (0–8.2)	0 (0–0.2)	<0.001**
Number ART naive	320 (40.3)	1460 (71)	

Body mass index (kg/m^2^)			
Mean (±SD)	24 (±3.4)	21 (±3.4)	<0.001
Missing	528 (66.5)	1016 (49.5)	

Total cholesterol mmol/L			
Mean (±SD)	5.15 (±1.3)	4.88 (±1.4)	<0.001

Triglycerides mmol/L			
Mean (±SD)	2.44 (±2)	2.40 (±2.3)	0.678

HDL-C mmol/L			
Mean (±SD)	1.15 (±0.5)	1.25 (±0.4)	<0.001

*Comparison by *t*-test for continuous variables and the *χ* ^2^ test for noncontinuous variables. **Comparison by Wilcoxon rank-sum test. A: AHOD: Australian HIV Observational Database; T: TAHOD: TREAT Asia HIV Observational Database. cART: combinational antiretroviral therapy; HBV: hepatitis B coinfection; HCV: hepatitis C coinfection; IDU: Intravenous drug user; IQR: interquartile range; SD: standard deviation. ***Transgender participants were classified as males in the multivariable analyses.

**Table 2:** Number and frequency of total cholesterol measurements by type of regimen and cohort.

cART Regimen	AHOD No. of patients*	AHOD median (range) no. of measurements per patient	AHOD no. (%) of measurements	TAHOD no. of patients*	TAHOD median (range) no. of measurements per patient	TAHOD no. (%) of measurements
NRTIs (+TDF) + NVP	122	2 (1–6)	247 (10.8)	70	1 (1–4)	108 (1.9)
NRTIs (+TDF) + EFV	131	2 (1–4)	245 (10.7)	90	1 (1–4)	147 (2.6)
NRTIs + NVP	155	2 (1–6)	342 (14.9)	696	1 (1–8)	1157 (20.6)
NRTIs + EFV	97	2 (1–4)	211 (9.2)	684	2 (1–10)	1487 (26.5)
NRTIs (+TDF) + PI (+ATV)	140	1 (1–5)	266 (11.6)	20	1 (1–4)	28 (0.5)
NRTIs (+TDF) + PI	187	2 (1–7)	410 (17.9)	137	3 (1–6)	367 (6.5)
NRTIs + PI (+ATV)	78	2 (1–5)	178 (7.7)	232	3 (1–6)	610 (10.9)
NRTIs + PI	184	2 (1–6)	396 (17.2)	620	3 (1–12)	1698 (30.3)

Total	1094		2295 (100)	2548		5602 (100)

*Each patient could contribute to more than one regimen. AHOD: Australian HIV Observational Database; ATV: Atazanavir; EFV: Efavirenz; NNRTI: non- nucleoside reverse transcriptase inhibitors; NRTIs: nucleoside reverse transcriptase inhibitors; NVP: nevirapine; PI: protease inhibitor; TAHOD: TREAT Asia HIV Observational Database; TDF: tenofovir.* Key*. NRTIs are TDF based (NRTIs + TDF) or not (NRTIs) and PI as ATV based (PI + ATV) or not (PI).

**Table 3:** Adjusted analyses for triglycerides, HDL-C, and total cholesterol : HDL-C ratio.**

Covariate	Mean difference in triglycerides mmol/L (95% CI)	Mean difference in HDL-C mmol/L (95% CI)	Mean difference in total cholesterol : HDL-C ratio (95% CI)
Regimen			
NRTIs (+TDF) + NVP	Reference	Reference	Reference
NRTIs (+TDF) + EFV	0.17 (−0.19 to 0.53)	0.01 (−0.06 to 0.08)	0.16 (−0.16 to 0.47)
NRTIs + NVP	0.58 (0.29 to 0.87)	0.15 (0.08 to 0.21)	0.13 (−0.12 to 0.39)
NRTIs + EFV	0.64 (0.34 to 0.95)	0.09 (0.03 to 0.16)	0.29 (0.01 to 0.56)
NRTIs (+TDF) + PI(+ATV)	0.15 (−0.22 to 0.52)	−0.03 (−0.11 to 0.05)	0.29 (−0.04 to 0.62)
NRTIs (+TDF) + PI	1.06 (0.73 to 1.38)	−0.02 (−0.09 to 0.05)	0.66 (0.37 to 0.95)
NRTIs + PI(+ATV)	0.57 (0.24 to 0.90)	0.03 (−0.05 to 0.10)	0.34 (0.02 to 0.65)
NRTIs + PI	1.13 (0.83 to 1.43)	0.01 (−0.06 to 0.07)	0.75 (0.47 to 1.03)
*P*	<0.001	<0.001	<0.001
Cohort			
AHOD	Reference	Reference	Reference
TAHOD	0.33 (0.11 to 0.55)	0.04 (−0.01 to 0.09)	−0.16 (−0.40 to 0.08)
*P*	0.003	0.146	0.187

Table shows independent effects of regimen and cohort variables in adjusted analyses. Since the interaction term between these variables was not significant, it was not included in the models.  Figure 1 shows the interaction between regimen and cohort variables.

**Multivariable models were *a priori* adjusted for time on given regimen, Hepatitis B and/or C coinfections, age, gender, HIV RNA viral load copies/mL, CD4+ T-cell count, BMI, cumulative exposure to cART at the start of regimen, and HIV exposure category. AHOD: Australian HIV Observational Database, ATV: Atazanavir; cART: combinational antiretroviral therapy; CI: confidence interval; EFV: Efavirenz; HBV: Hepatitis B co-infection; HCV: Hepatitis C co-infection; IDU: Intravenous drug user; NNRTI: nonnucleoside reverse transcriptase inhibitors; NRTI: nucleoside reverse transcriptase inhibitors, NVP: nevirapine, PI: protease inhibitor, TAHOD: TREAT Asia HIV Observational Database; TDF: Tenofovir. *Key*. NRTIs are TDF based (NRTIs + TDF) or not (NRTIs) and PI as ATV based (PI + ATV) or not (PI).
